# Asthma–COPD overlap syndrome (ACOS) in primary care of four Latin America countries: the PUMA study

**DOI:** 10.1186/s12890-017-0414-6

**Published:** 2017-04-21

**Authors:** Maria Montes de Oca, Maria Victorina Lopez Varela, Maria E. Laucho-Contreras, Alejandro Casas, Eduardo Schiavi, Juan Carlos Mora

**Affiliations:** 1 Servicio de Neumonología, Hospital Universitario de Caracas, Facultad de Medicina, Los Chaguaramos, 1030, Universidad Central de Venezuela, Caracas, Venezuela; 2 Universidad de la República, Facultad de Medicina, Hospital Maciel, Montevideo, Uruguay; 3 Fundación Neumológica Colombiana, Bogotá, Colombia; 4 Hospital de Rehabilitación Respiratoria María Ferrer, Buenos Aires, Argentina; 5 AstraZeneca Medical Department, Medellín, Colombia

**Keywords:** ACOS, Latin America, Prevalence, PUMA

## Abstract

**Background:**

Asthma–COPD overlap syndrome (ACOS) prevalence varies depending on the studied population and definition criteria. The prevalence and clinical characteristics of ACOS in an at-risk COPD primary care population from Latin America was assessed.

**Methods:**

Patients ≥40 years, current/ex-smokers and/or exposed to biomass, attending routine primary care visits completed a questionnaire and performed spirometry. COPD was defined as post-bronchodilator forced expiratory volume in 1 s/forced vital capacity (FEV_1_/FVC) < 0.70; asthma was defined as either prior asthma diagnosis or wheezing in the last 12 months plus reversibility (increase in post-bronchodilator FEV_1_ or FVC ≥200 mL and ≥12%); ACOS was defined using a combination of COPD with the two asthma definitions. Exacerbations in the past year among the subgroups were evaluated.

**Results:**

One thousand seven hundred forty three individuals completed the questionnaire, 1540 performed acceptable spirometry, 309 had COPD, 231 had prior asthma diagnosis, and 78 asthma by wheezing + reversibility. ACOS prevalence in the total population (by post-bronchodilator FEV_1_/FVC < 0.70 plus asthma diagnosis) was 5.3 and 2.3% by post-bronchodilator FEV_1_/FVC < 0.70 plus wheezing + reversibility. In the obstructive population (asthma or COPD), prevalence rises to 17.9 and 9.9% by each definition, and to 26.5 and 11.3% in the COPD population. ACOS patients defined by post-bronchodilator FEV_1_/FVC < 0.7 plus wheezing + reversibility had the lowest lung function measurements. Exacerbations for ACOS showed a prevalence ratio of 2.68 and 2.20 (crude and adjusted, *p* < 0.05, respectively) (reference COPD).

**Conclusions:**

ACOS prevalence in primary care varied according to definition used. ACOS by post-bronchodilator FEV_1_/FVC < 0.7 plus wheezing + reversibility represents a clinical phenotype with more frequent exacerbations, which is probably associated with a different management approach.

**Electronic supplementary material:**

The online version of this article (doi:10.1186/s12890-017-0414-6) contains supplementary material, which is available to authorized users.

## Background

Both chronic obstructive pulmonary disease (COPD) and asthma are common chronic airway diseases that contribute to morbidity and mortality in adults worldwide. The coexistence of these two pathologies in some individuals is recognised as asthma–COPD overlap syndrome (ACOS). The prevalence of this phenotype varies considerably between different studies and this is primarily related to the heterogeneity of the criteria used to define asthma and COPD, and the population being studied (e.g. general population, asthma, COPD).

The prevalence of ACOS in the total population ranges from 1.6 to 4.5% in different studies around the world [1–5]. If only subjects with asthma or COPD are included, the prevalence of ACOS among patients with COPD ranges from 12.1 to 55.2%, and among patients with asthma from 13.3 to 61.0% [1–19]. The wide variation in prevalence is related to the diagnostic criteria applied when defining asthma (self-reported physician diagnosis vs. clinical and/or spirometry-based diagnosis) and COPD (self-reported physician diagnosis vs. spirometric criteria: forced expiratory volume in 1 s/forced vital capacity [FEV_1_/FVC] <0.70), together with the population being studied.

Little is known regarding the prevalence of ACOS in Latin America. The Latin American Project for the Investigation of Lung Disease (PLATINO) population-based study showed that two different definitions of asthma resulted in varied ACOS prevalence estimates in the same population [3]. The prevalence of ACOS based on post-bronchodilator FEV_1_/FVC <0.70 and the presence of wheezing in the last year plus reversibility was estimated to be 1.8%, compared with 2.9% when using post-bronchodilator FEV_1_/FVC <0.70 and physician diagnosis of asthma [3].

Data from two recent systematic reviews suggest that ACOS is associated with more frequent adverse outcomes than either asthma or COPD. ACOS patients have shown higher healthcare utilisation, higher exacerbation rates, more symptoms and lower health-related quality of life (HRQOL) [20, 21]. However, in contrast to this, results from the COPD History Assessment In SpaiN (CHAIN) study showed that survival after 1 year of follow-up was better in ACOS patients than in clinically similar patients with COPD without any ACOS criteria. In addition, the authors reported that this phenotype was not associated with any other baseline clinical differences or worse clinical outcomes [22].

To our knowledge, limited information exists on the prevalence and clinical characteristics of ACOS phenotype in primary care [23]. Therefore, the aims of this study were to measure the prevalence of ACOS using different definitions in an at-risk COPD population (≥40 years) attending primary care settings in four Latin American countries, to assess the clinical characteristics of these subjects, and to determine the association between ACOS and the following clinical outcomes: exacerbation, hospitalisation and dyspnoea severity.

## Methods

The Prevalence StUdy and Regular Practice, Diagnosis and TreatMent, Among General Practitioners in Populations at Risk of COPD in Latin America (PUMA) study was conducted in the primary care setting of four Latin American countries: Argentina, Colombia, Venezuela, and Uruguay. Complete details of the methodology have been published previously [24–27]. In summary, this was a multicentre, multinational, cross-sectional, non-interventional study. Participating sites were selected according to local feasibility based on a previous local availability database of potential principal investigators (not randomised) and included primary care centres (family doctors, general practitioners etc.) with no direct connection with respiratory medicine specialists. These sites were selected to reflect the reality of national primary care practice in terms of geographical distribution and healthcare sector. The ethics committees for each site involved in the study approved the protocol and all participants provided written informed consent.

At-risk patients were included in the study if they were ≥40 years of age, current or ex-smokers (≥10 pack-years, ≥50 pipes/year or ≥50 cigars/year) [28] and/or exposed to biomass smoke (wood or coal for cooking and heating; exposure ≥100 h/year) [29, 30].

Participants completed a modified version of the PLATINO study questionnaire [31] for information on factors associated with COPD; these included demographics, smoking habits, education, employment, respiratory symptoms that included a question on wheezing in the last 12 months, comorbidities, use of respiratory medication and prior spirometric testing. Data on prior medical diagnosis of tuberculosis, asthma, chronic bronchitis, emphysema, and COPD were also obtained. A comorbidity score was calculated [32]. Spirometry was performed using the portable, battery-operated ultrasound Easy One spirometer (ndd Medical Technologies, Zurich, Switzerland). Spirometry tests were performed at baseline and 15 min after the inhalation of 400 μg salbutamol, according to the American Thoracic Society (ATS) criteria of acceptability and reproducibility [33].

For the purpose of this study, the following definitions of asthma, COPD and ACOS were used:

1. COPD: A ratio of post-bronchodilator FEV_1_/FVC <0.70 (GOLD definition) [34].
2. Asthma: Two definitions were used: (A) medical diagnosis of asthma (self-reported prior medical diagnosis); (B) the presence of wheezing in the last 12 months plus reversibility (post-bronchodilator increase in FEV_1_ or FVC of 200 mL and 12%).
3. ACOS: Two definitions of ACOS validated in a previous study were used in the present study [3]. The combination of the COPD definition above with both asthma criteria separately: (A) a ratio of post-bronchodilator FEV_1_/FVC <0.70 plus prior medical diagnosis of asthma; (B) a ratio of post-bronchodilator FEV_1_/FVC <0.70 and wheezing in the last 12 months plus reversibility (post-bronchodilator increase in FEV_1_ or FVC of 200 mL and 12%).
  Severity of COPD airway obstruction and disease stratification were determined using the GOLD criteria [34].

### Outcomes

Dyspnea by the mMRC scale and COPD exacerbation among the subgroups were evaluated. COPD exacerbations were self-reported and defined by deterioration of breathing symptoms that affected usual daily activities or caused absences from work. We examined the proportion of subjects in each group who reported: 1) any exacerbation within the previous 12-months; 2) an exacerbation requiring a hospitalisation within the previous 12-months. We also examined the number of the exacerbation-related events within the previous 12-months.

### Statistical analysis

Descriptive statistics were calculated using absolute and relative frequencies for categorical variables and means (median) and standard deviation (interquartile range) for numerical ones. For comparisons among numerical variables an ANOVA was used and a chi-squared test was used for comparisons between categorical variables. A *p*-value of <0.05 was considered statistically significant. Crude and adjusted Poisson regression models were performed in order to obtain the prevalence ratio for outcomes and each independent variable. A Wald test for heterogeneity or for trend (in specific cases) was considered. All analyses were performed using Stata 13.0 statistical software.

## Results

**Fig. 1:**
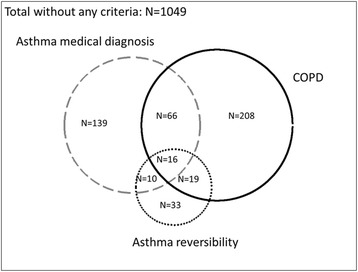
Venn diagram showing the three phenotypes and the overlap in the PUMA study

A total of 1743 individuals completed study questionnaire and 1540 performed acceptable spirometry. Figure 1 is a Venn diagram displaying the overlap of the different diagnoses among these subjects. In the total PUMA study population, 1049 subjects did not have asthma, COPD or ACOS. Based on post-bronchodilator FEV_1_/FVC <0.70 criteria, COPD was present in 309 patients; 231 patients had a medical diagnosis of asthma and 78 patients had asthma defined by reversibility plus wheezing. ACOS, defined as an asthma medical diagnosis and COPD was present in 82 patients, and defined by reversibility plus wheezing and COPD in 35 patients (Fig. 1).

**Fig. 2:**
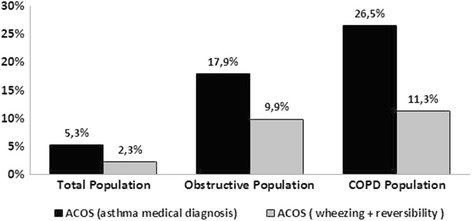
ACOS prevalence using the different definitions (post-bronchodilator FEV_1_/FVC <0.70 plus wheezing + reversibility, and post-bronchodilator FEV_1_/FVC <0.70 plus medical diagnosis of asthma) in different populations: total population, obstructive population (asthma + COPD), or COPD population

Figure 2 shows the prevalence of ACOS according to different definitions used in the different populations (i.e. the denominator used when calculating prevalence). As expected, ACOS prevalence depends on the population (denominator) chosen: total study population, obstructive population (those affected with either asthma or COPD) or COPD population. The prevalence of ACOS in the total study population defined as asthma medical diagnosis plus FEV_1_/FVC <0.70 was higher (5.3%; 82/1540 subjects) than when using the reversibility plus wheezing and FEV_1_/FVC <0.70 definition (2.3%; 35/1540 subjects). A similar trend in ACOS prevalence was found in the obstructive population (82/458 subjects, 17.9% by asthma medical diagnosis and FEV_1_/FVC <0.70 definition; and 35/352 subjects, 9.9% by reversibility plus wheezing and FEV_1_/FVC <0.70 definition) and the COPD population (82/309 subjects, 26.5% by asthma medical diagnosis and FEV_1_/FVC <0.70 definition; and 35/309 subjects, 11.3% by reversibility plus wheezing and FEV_1_/FVC <0.70 definition).

The characteristics of subjects with COPD, asthma and ACOS according to the definition of COPD and asthma (wheezing + reversibility) are shown in Table 1. Using these definitions, there were no differences in biomass smoke exposure or comorbidities between the groups. However, subjects in the asthma group were younger, predominantly female, smoked less and had the highest body mass index. Those in the ACOS group, compared with individuals in the COPD group, were of similar in age, gender (predominantly male), body mass index and pack-years of smoking. The ACOS group had the highest percentage of symptoms (cough, phlegm and dyspnoea), self-reported diagnosis of asthma, exacerbations and hospitalisation due to exacerbation within the past year. A similar distribution of subjects according to GOLD spirometry stage was observed for the COPD and ACOS groups. However, using the new GOLD 2013 staging system (A–D), the ACOS group had a greater proportion of patient categorised as C and D (40%) compared with the COPD group (30%) (Table 1). Similar findings to those reported above were observed when the asthma medical diagnosis and FEV_1_/FVC <0.70 definition for ACOS was used (Table 2).

**Table 1:** Characteristics of subjects with COPD (post-bronchodilator FEV_1_/FVC <0.70), asthma (wheezing + reversibility) and ACOS (post-bronchodilator FEV_1_/FVC <0.70 plus wheezing + reversibility)

Variables	Asthma	COPD	ACOS	*p*-value
	(*N* = 43)	(*N* = 274)	(*N* = 35)	
Age, years, mean (SD)	56.3 (8.9)	67.3 (9.4)	65.2 (8.6)	<0.001
Age, complete years, n (%)				<0.001
40–49	10 (23.3)	6 (2.2)	1 (2.9)	
50–59	19 (44.2)	61 (22.3)	10 (28.6)	
≥ 60	14 (32.5)	20 (75.6)	24 (68.6)	
Gender male, n (%)	20 (46.5)	150 (54.7)	23 (65.7)	0.029
BMI, kg/m^2^, mean (SD)	29.9 (6.2)	26.4 (6.0)	26.0 (4.9)	0.009
BMI, kg/m^2^, n (%)				0.015
< 25	10 (23.3)	125 (45.6)	13 (37.1)	
25–29.9	15 (34.9)	93 (33.9)	14 (40.0)	
≥ 30	18 (41.9)	56 (20.4)	8 (22.9)	
Smoking, pack-years, mean (SD)	27.2 (18.7)	44.3 (28.9)	48.9 (37.7)	<0.001
Pack-years smoked during life, n (%)				<0.001
< 20	16 (37.2)	49 (18.4)	5 (14.7)	
20–30	15 (34.9)	42 (15.8)	6 (17.7)	
> 30	12 (27.9)	175 (65.8)	23 (67.6)	
Biomass exposure, complete years, n (%)				0.501
≥ 10	16 (37.2)	113 (41.2)	11 (31.4)	
Smoking status, n (%)				0.728
Never	2 (4.7)	4 (1.5)	1 (2.9)	
Former	23 (53.5)	158 (58.3)	20 (57.1)	
Current	18 (41.9)	109 (40.2)	14 (40.0)	
Respiratory symptoms present, n (%)				
Cough	15 (34.9)	115 (42.0)	10 (57.1)	<0.001
Phlegm	21 (48.8)	116 (42.3)	23 (65.7)	<0.001
Wheezing	43 (100.0)	39 (14.2)	35 (100.0)	<0.001
Dyspnoea	17 (46.0)	156 (61.7)	26 (78.8)	<0.001
mMRC scale, mean (SD)	0.9 (1.2)	1.4 (1.3)	1.7 (1.3)	0.035
Prior spirometry, n (%)	9 (20.9)	98 (35.8)	16 (45.7)	<0.001
Self-reported diagnosis: COPD, n (%)	1 (2.3)	63 (23.0)	8 (22.9)	<0.001
Self-reported diagnosis: Asthma, n (%)	10 (23.3)	66 (24.1)	16 (45.7)	<0.001
Comorbidity score, mean (SD)	1.1 (1.1)	1.2 (1.0)	0.9 (0.8)	0.538
Comorbidity score, n (%)				0.067
None	12 (27.9)	61 (22.6)	13 (37.1)	
1	22 (51.2)	113 (41.9)	11 (31.4)	
2	5 (11.6)	70 (25.9)	11 (31.4)	
3+	4 (9.3)	26 (9.6)	-	
Any exacerbation within the past year, n (%) (Yes)	7 (16.3)	25 (9.1)	8 (22.9)	<0.001
Number of exacerbations, past year, mean (SD)	0.4 (1.0)	0.2 (0.8)	0.4 (1.0)	0.002
Hospitalisation due to exacerbation, past year, n (%)	1 (2.3)	8 (2.9)	3 (8.6)	<0.001
GOLD 2007 stage, n (%)				<0.001
No	43 (100.0)	-	-	
1	-	50 (18.3)	3 (8.6)	
2	-	148 (54.0)	21 (60.0)	
3	-	56 (20.4)	8 (22.9)	
4	-	20 (7.3)	3 (8.6)	
GOLD 2013 stage, n (%)				<0.001
No	43 (100.0)	-	-	
A	-	120 (43.8)	13 (37.1)	
B	-	71 (25.9)	8 (22.9)	
C	-	24 (8.8)	4 (11.4)	
D	-	59 (21.5)	10 (28.6)	

*Abbreviations*: *BMI* Body mass index

Maximum number of missing for each category of ACOS is for dyspnoea (asthma *n* = 6, COPD *n* = 21 and ACOS *n* = 2)

**Table 2:** Characteristics of subjects with COPD (post-bronchodilator FEV_1_/FVC <0.70), asthma (prior medical diagnosis of asthma) and ACOS (post-bronchodilator FEV_1_/FVC <0.70 and prior medical diagnosis of asthma)

Variables	Asthma	COPD	ACOS	*p*-value
	(*N* = 149)	(*N* = 227)	(*N* = 82)	
Age, years, mean (SD)	56.3 (9.7)	67.8 (9.0)	65.0 (9.8)	<0.001
Age, complete years, n (%)				<0.001
40–49	42 (28.2)	4 (1.8)	3 (3.7)	
50–59	60 (40.3)	44 (19.4)	27 (32.9)	
≥ 60	47 (31.5)	179 (78.8)	52 (63.4)	
Gender, male, n (%)	43 (28.9)	134 (59.0)	39 (47.6)	<0.001
BMI, kg/m^2^, mean (SD)	30.0 (5.5)	26.3 (5.8)	26.4 (6.1)	<0.001
BMI, kg/m^2^, n (%)				<0.001
< 25	25 (16.8)	104 (45.8)	34 (41.5)	
25–29.9	55 (36.9)	75 (33.0)	32 (39.0)	
≥ 30	69 (46.3)	48 (21.2)	16 (19.5)	
Smoking, pack-years, mean (SD)	26.8 (21.6)	48.3 (30.2)	35.0 (27.4)	<0.001
Pack-years smoked during life, n (%)				<0.001
< 20	70 (49.7)	27 (12.2)	27 (34.6)	
20–30	23 (16.3)	36 (16.2)	12 (15.4)	
> 30	48 (34.0)	159 (71.6)	39 (50.0)	
Biomass exposure, complete years, n (%)				0.004
≥ 10	38 (25.5)	96 (42.3)	28 (34.1)	
Smoking status, n (%)				0.001
Never	11 (7.4)	2 (0.9)	3 (3.8)	
Former	93 (62.8)	134 (59.3)	44 (55.0)	
Current	44 (29.7)	90 (39.8)	33 (41.3)	
Respiratory symptoms present, n (%)				
Cough	56 (37.6)	95 (41.9)	40 (48.8)	<0.001
Phlegm	40 (26.9)	100 (44.1)	39 (47.6)	<0.001
Wheezing	40 (26.9)	48 (21.2)	26 (31.7)	<0.001
Dyspnoea	83 (61.0)	126 (59.4)	56 (75.7)	<0.001
mMRC scale, mean (SD)	1.1 (1.3)	1.3 (1.3)	1.7 (1.3)	0.166
Prior spirometry, n (%)	48 (32.2)	66 (29.1)	48 (58.5)	<0.001
Self-reported diagnosis: COPD, n (%)	5 (3.4)	46 (20.3)	25 (30.5)	<0.001
Self-reported diagnosis: Asthma, n (%)	149 (100.0)	0 (0.0)	82 (100.0)	<0.001
Comorbidity score, mean (SD)	1.2 (1.0)	1.2 (0.9)	1.1 (1.0)	0.013
Comorbidity score, n (%)				0.742
None	39 (26.2)	50 (22.3)	24 (29.6)	
1	57 (38.3)	92 (41.1)	32 (39.5)	
2	36 (24.2)	63 (28.1)	18 (22.2)	
3+	17 (11.4)	19 (8.5)	7 (8.6)	
Any exacerbation within the past year, n (%) (Yes)	23 (15.4)	19 (8.4)	14 (17.1)	<0.001
Number of exacerbations, past year, mean (SD)	0.4 (1.0)	0.2 (0.7)	0.4 (0.9)	<0.001
Hospitalisation due to exacerbation, past year, n (%)	3 (2.0)	8 (3.5)	3 (3.7)	0.006
GOLD 2007 stage, n (%)				<0.001
No	149 (100.0)	-	-	
I	-	43 (18.9)	10 (12.2)	
II	-	124 (54.6)	45 (54.9)	
III	-	44 (19.4)	20 (24.4)	
IV	-	16 (7.1)	7 (8.5)	
GOLD 2013 stage, n (%)				<0.001
No	149 (100.0)	-	-	
A	-	104 (45.8	29 (35.4)	
B	-	57 (25.1)	22 (26.8)	
C	-	18 (7.9)	10 (12.2)	
D	-	48 (21.2)	21 (25.6)	

*Abbreviations*: *BMI* Body mass index

Maximum number of missing for each category of ACOS is for dyspnoea (asthma *n* = 13, COPD *n* = 15 and ACOS *n* = 8)

When comparing the three groups, the ACOS patients (defined by wheezing plus reversibility and FEV_1_/FVC <0.70) had the lowest lung function measurements for pre- and post-bronchodilator FEV_1_ and FVC (Table 3). The ACOS patients had a higher reversibility (% change) for FEV_1_ and FVC compared with the other two groups (Table 3). Again, similar findings were also found when the other ACOS definition was used (medical diagnosis of asthma and FEV_1_/FVC <0.70) (Table 4).

**Table 3:** Lung function parameters of subjects with COPD (post-bronchodilator FEV_1_/FVC <0.70), asthma (wheezing + reversibility) and ACOS (post-bronchodilator FEV_1_/FVC <0.70 plus wheezing + reversibility)

Variables	Asthma	COPD	ACOS	*P*-value
	(*N* = 43)	(*N* = 274)	(*N* = 35)	
Pre-bronchodilator FEV_1_, L	2.3 (0.7)	1.6 (0.7)	1.4 (0.6)	<0.001
Pre-bronchodilator FEV_1_, % pred.	84.3 (17.2)	61.8 (22.6)	50.7 (17.3)	<0.001
Post-bronchodilator FEV_1_, L	2.6 (0.7)	1.7 (0.7)	1.6 (0.6)	<0.001
Post-bronchodilator FEV_1_, % pred.	93.1 (15.8)	64.7 (21.1)	58.8 (18.6)	<0.001
FEV_1_ change, mL (absolute)	237.7 (131.7)	73.7 (176.7)	215.4 (133.7)	<0.001
FEV_1_ change, % (relative)	11.7 (10.1)	8.1 (28.9)	17.5 (13.3)	<0.001
Pre-bronchodilator FVC, L	3.0 (0.9)	2.6 (0.9)	2.5 (0.8)	<0.001
Pre-bronchodilator FVC, % pred.	84.0 (17.0)	75.2 (19.3)	66.7 (17.7)	<0.001
Post-bronchodilator FVC, L	3.3 (0.9)	2.7 (0.9)	2.9 (0.8)	<0.001
Post-bronchodilator FVC, % pred.	91.2 (15.4)	79.0 (18.8)	76.3 (17.7)	<0.001
FVC change, mL (absolute)	244.9 (151.6)	135.0 (238.0)	346.6 (173.4)	<0.001
FVC change, % (relative)	9.8 (9.5)	6.2 (11.4)	15.6 (9.3)	<0.001

Values are presented as mean (standard deviation)

*Abbreviations*: *FEV*_1_ forced expiratory volume in 1 s, *FVC* forced vital capacity

**Table 4:** Lung function parameters of subjects with COPD (post-bronchodilator FEV_1_/FVC <0.70), asthma (prior medical diagnosis of asthma) and ACOS (post-bronchodilator FEV_1_/FVC <0.70 plus prior medical diagnosis of asthma)

Variables	Asthma	COPD	ACOS	*p*-value
	(*N* = 149)	(*N* = 227)	(*N* = 82)	
Pre-bronchodilator FEV_1_, L	2.4 (0.6)	1.6 (0.7)	1.4 (0.6)	<0.001
Pre-bronchodilator FEV_1_, % pred.	90.0 (16.3)	62.7 (22.1)	54.6 (21.9)	<0.001
Post-bronchodilator FEV_1_, L	2.4 (0.6)	1.7 (0.7)	1.5 (0.6)	<0.001
Post-bronchodilator FEV_1_, % pre.	92.3 (14.2)	65.2 (20.9)	60.7 (20.7)	<0.001
FEV_1_ change, mL (absolute)	56.7 (173.8)	68.1 (169.6)	149.6 (187.8)	<0.001
FEV_1_ change, % (relative)	3.5 (10.5)	6.0 (13.4)	17.9 (48.2)	<0.001
Pre-bronchodilator FVC, L	3.0 (0.7)	2.7 (0.9)	2.4 (0.8)	<0.001
Pre-bronchodilator FVC, % pred.	88.4 (15.6)	75.7 (19.2)	70.1 (19.1)	<0.001
Post-bronchodilator FVC, L	3.0 (0.7)	2.8 (0.9)	2.6 (0.8)	<0.001
Post-bronchodilator FVC, % pred.	89.1 (13.9)	79.4 (18.6)	76.6 (18.8)	<0.001
FVC change, mL (absolute)	21.8 (200.5)	139.1 (249.3)	214.0 (207.6)	<0.001
FVC change, % (relative)	1.6 (9.6)	6.2 (11.8)	10.3 (10.3)	<0.001

Values are presented as mean (standard deviation)

*Abbreviations*: *FEV*_1_ forced expiratory volume in 1 s, *FVC* forced vital capacity

Table 5 shows the prevalence ratio and relative risk (crude results and adjusted analysis) for the different phenotypes according to the presence of exacerbations, number of exacerbations, hospitalisations due to exacerbation in the past year and mMRC scale. In the ACOS group defined as wheezing plus reversibility and FEV_1_/FVC <0.70 the presence of exacerbations showed crude and adjusted prevalence ratios of 2.68 and 2.20 (COPD as reference group), respectively. The number of exacerbations was not statistically significant for ACOS group (COPD as reference group). The prevalence ratio for hospitalisations and mMRC scale among phenotypes by this definition or when using the asthma medical diagnosis and FEV_1_/FVC <0.70 definition were not statistically significant (COPD as reference group). The regression coefficient crude and adjusted analysis for all variables in model * + FEV_1_ (absolute values, ml), for all variables in model * + FEV_1_ (absolute values, ml) + height, for all variables in model * + FEV_1_ (% predicted according to PLATINO equation) and for all variables in model * + GOLD stages in the different phenotypes is shown in Additional file 1: Table S1.

**Table 5:** Prevalence ratio and relative risk (crude and adjusted analysis) for exacerbations, hospitalisations due to exacerbation in the past year and mMRC scale in the different phenotypes

	Asthma	*p*-value	COPD	ACOS	*p*-value
*Asthma defined by wheezing + reversibility*					
Exacerbations in the past year (yes/no)					
Unadjusted – PR (95% CI)	1.85 (0.85; 4.06)	*0.122*	1.00	2.68 (1.30; 5.52)	*0.007*
Adjusted^a^– PR (95% CI)	2.24 (0.92; 5.45)	*0.075*	1.00	2.20 (1.10; 4.39)	*0.026*
Number of exacerbations in the past year					
Unadjusted – RR (95% CI)	1.77 (0.72; 4.34)	*0.210*	1.00	2.10 (0.90; 4.90)	*0.086*
Adjusted^a^– RR (95% CI)	2.84 (0.94; 8.61)	*0.065*	1.00	1.64 (0.78; 3.44)	*0.191*
Hospitalisations in the past year					
Unadjusted – PR (95% CI)	0.76 (0.10; 5.96)	*0.795*	1.00	2.89 (0.80; 10.39)	*0.104*
Adjusted^a^– PR (95% CI)	3.57 (0.48; 26.59)	*0.214*	1.00	1.65 (0.53; 5.06)	*0.385*
mMRC scale					
Unadjusted – RR (95% CI)	0.64 (0.41; 0.99)	*0.289*	1.00	1.17 (0.88; 1.56)	*0.046*
Adjusted^a^– RR (95% CI)	0.73 (0.48; 1.12)	*0.149*	1.00	1.22 (0.92; 1.12)	*0.176*
*Asthma defined as medical diagnosis*					
Exacerbations in the past year (yes/no)					
Unadjusted – PR (95% CI)	1.80 (1.01; 3.20)	*0.046*	1.00	1.80 (0.91; 3.53)	*0.089*
Adjusted^a^– PR (95% CI)	1.57 (0.75; 3.27)	*0.231*	1.00	1.29 (0.64; 2.60)	*0.480*
Number of exacerbations in the past year					
Unadjusted – RR (95% CI)	1.92 (0.99; 3.68)	*0.054*	1.00	1.68 (0.77; 3.66)	*0.191*
Adjusted^a^– RR (95% CI)	2.01 (0.94; 4.30)	*0.072*	1.00	1.32 (0.60; 2.88)	*0.490*
Hospitalisations in the past year					
Unadjusted – PR (95% CI)	0.58 (0.16; 2.16)	*0.419*	1.00	1.07 (0.29; 3.92)	*0.923*
Adjusted^a^– PR (95% CI)	0.68 (0.17; 2.69)	*0.581*	1.00	0.72 (0.21; 2.44)	*0.596*
mMRC scale					
Unadjusted – RR (95% CI)	0.90 (0.72; 1.14)	*0.311*	1.00	1.24 (0.98; 1.56)	*0.052*
Adjusted^a^– RR (95% CI)	0.97 (0.75; 1.25)	*0.799*	1.00	1.20 (0.96; 1.21)	*0.108*

^a^Adjusted for age, sex, skin colour, body mass index, schooling, comorbidity score, pack-years and any treatment (bronchodilator or corticosteroid)

*Abbreviations*: *PR* prevalence ratio, *RR* relative risk

## Discussion

The principal findings of this study are: first, ACOS prevalence depends on the asthma component definition and the population it is being evaluated in (e.g. general population, obstructive population or COPD population). The lowest prevalence was 2.3% when ACOS was defined as wheezing plus reversibility and FEV_1_/FVC <0.70 in the total population, whereas the highest was 26.5% when ACOS was defined as previous medical diagnosis of asthma and FEV_1_/FVC <0.70 in the COPD population. Second, after adjusting for confounding factors, ACOS defined as FEV_1_/FVC <0.7 and wheezing plus reversibility was associated with a higher risk for exacerbations compared with those subjects with COPD.

Proposed definitions for ACOS vary widely and include: a) patients with COPD who have a previous diagnosis of asthma; b) patients with a spirometric COPD definition who have significant reversibility (FEV_1_/FVC <0.70 and post-bronchodilator increase in FEV_1_ or FVC of 200 mL and 12%); c) patients with asthma who have persistent airflow obstruction. It is important to recognise whether a patient has ACOS as it may influence the clinical course, long-term outcome, and response to therapy. Other documents such as the Global Initiative for Asthma (GINA)–Global Initiative for Chronic Obstructive Lung Disease (GOLD) consensus and the Spanish guideline for COPD have also proposed their own definitions [35, 36], however they have not been fully validated in large cohorts.

Using a definition similar to the present study, some authors have assessed ACOS prevalence in the general population. Marsh et al. found a prevalence of ACOS of 11% in the total population studied and 55% in the COPD population [18]. However, this study was conducted only in volunteers and had a small sample size. Using the population-based Spanish EPI-SCAN study data, Miravitlles et al. reported a prevalence of ACOS in the general population of 1.7%, and of 17.4% in the COPD patients using the previous asthma diagnosis definition [19].

The PLATINO study reports a prevalence based on previous asthma diagnosis and FEV_1_/FVC <0.70 criteria in the total population of 2.9%, and a prevalence of 1.8% using criteria of wheezing plus reversibility and FEV_1_/FVC <0.70 [3]. In the same study, in the obstructive population, the prevalence was 13% by previous asthma diagnosis and FEV_1_/FVC <0.70 criteria, and 11.6% by wheezing plus reversibility and FEV_1_/FVC <0.70 [3]. Other authors have assessed the prevalence of ACOS in selected COPD populations [6, 7, 22, 37]. The prevalence of ACOS in the COPDGene study was 12.6% using self-reported asthma criteria [7], and similar results have been reported elsewhere [6]. Recently, Cosio et al. reported an ACOS prevalence of 15% in a COPD Spanish cohort of over 800 patients using one major criterion for asthma definition (reversibility >400 mL and 15% plus medical history of asthma) or two minor criteria (blood eosinophils >5%, IgE >100 IU/mL, or two separate bronchodilator tests >200 mL and 12%) [22]. A higher prevalence (25%) was reported in the ECLIPSE cohort when using their primary study definition of COPD patients answering “yes” to the question “Have you ever had asthma?” [37].

Little information exists regards the prevalence of ACOS in the primary care setting. As expected, the prevalence varied depending on the method by which ACOS was defined. Barrechenguren et al. reported a prevalence of 5.4% using the previous diagnosis of asthma in newly diagnosed patients with COPD [38]. In a separate study, the same authors found a higher ACOS prevalence in COPD patients with a history of asthma (10.8%) [39]. Others have reported a prevalence of 5.5% using a history of asthma in the total study population, and 19.1% using a restrictive analysis (asthma defined by reversibility criteria) in the COPD population [40].

The findings of the present study are consistent with those reported in some general and selected COPD populations that have used the previous diagnosis of asthma plus spirometric COPD to define ACOS [3, 5, 7, 30]. The comparison with the PLATINO study deserves special consideration as this is another study from Latin America that use the same two ACOS definitions in the same population [3]. The most important difference between the two studies that needs to be highlighted is that the PLATINO study was a larger population-based (general population) study, whereas PUMA is a study in a primary care population at risk for COPD; as a result of these being two different populations, differences in the results are to be expected. The prevalence of ACOS by both definitions reported here in the PUMA study (population at risk for COPD) were slightly higher than those reported in the PLATINO study.

The above-mentioned findings support the concept that the criteria used to define ACOS, as well as the population used to calculate the prevalence, have a significant influence on prevalence; it is thus essential to know this information when interpreting the results of other studies. The discrepancies observed with the findings of primary care studies could be partially explained by the selection of participating patients (only newly diagnosed COPD patients and/or a younger population), and the ACOS definition used [38–40]. However, when spirometric COPD diagnosis and asthma defined by reversibility was used to define ACOS elsewhere [40], the prevalence was similar to our results in the COPD population. Another important aspect to highlight is that the Latin American population has a very distinct characteristic of being exposed to biomass fuel. There is no literature on biomass exposure and ACOS. In the present study, more than a third of the patients in each group had biomass exposure and irrespective of the definition used, approximately 3% of patients with ACOS had no smoking history. The size of the PUMA sample does not allow us to analyse the characteristics of ACOS patients due to biomass exposure; therefore, futures studies in regions with high biomass exposure, such as Latin America, aimed at characterizing this group of patients are warranted.

Two recent systematic reviews and meta-analyses indicate that ACOS patients may have more symptoms, more frequent exacerbations and hospitalisations, worse HRQOL and higher healthcare costs than patients with only asthma or COPD [20, 21]. Similar to patients with COPD, ACOS patients appear to have a high occurrence of comorbidities, including diabetes. In agreement with the results of these systematic reviews, we found the spirometry plus symptom-based (wheezing plus reversibility and FEV_1_/FVC <0.70) definition identify a clinical phenotype with more frequent exacerbations. Also, in agreement with other results, we did not find any difference in the number of comorbidities between the groups [39]. In the present study, the ACOS patients (defined by wheezing plus reversibility and FEV_1_/FVC <0.70) had the lowest lung function measurements. These findings are consistent with other studies in population-based sample that reported lower level of lung function in the ACOS subjects compared with asthma and COPD groups [3, 41]. As has been mentioned previously, there is no universal definition for ACOS. However, this is a phenotype recognised as a different COPD subpopulation with important therapeutic implications. The GINA–GOLD consensus recommends the use of inhaled corticosteroids (ICS) in patients with suspected ACOS [34]. However, ICS therapy has been linked with increased risk of pneumonia in COPD patients [42, 43], so it is crucial to be as accurate as possible with the prevalence of ACOS as well as determining the most appropriate definition to avoid over-diagnosis and subsequent overuse of ICS in patients with COPD.

Finally, when considering and interpreting the current findings, it is important to be aware of the following study limitations: these results are not generalizable to all Latin American countries as the study was only performed in four countries; it is possible that some results did not reach statistical significance as a result of sample size and lack of power, despite the efforts made to ensure a representative sample. Nevertheless, the procedure used was the most reasonable in view of the operational possibilities in each country; this was a transversal study and so was only designed to evaluate the characteristics of the patients and not the follow-up; we did not assess any pathophysiological link among ACOS, COPD and asthma, or a pathway that could explain the characteristics of the ACOS patients. It is important to note that the PUMA centres were not randomised, so sites selection did not follow a representative sampling of national primary care practice. In addition, other limitations to consider are that the diagnosis of asthma was, in part, based on patient recall and this may influence the true “incidence” of ACOS, and “wheezing” was obtained from questionnaires and was not directly observed by a physician. Finally, it is important to highlight that although wheezing is a hallmark of asthma, it often occurs in COPD, especially during exacerbations. Hence, if a patient has wheezing there is a possibility that this symptom originated from a COPD exacerbation; therefore, is not entirely surprising that patients with “ACOS” had higher incidence of exacerbations. Another limitation is the lack of a variable that could indicate severity. We performed a sensitivity analysis including FEV_1_ in the model as a proxy of severity, but the statistical model became unstable with variance inflation factors higher than 10. It should be highlighted that the direction of the association did not change adding FEV_1_. Therefore, we opted to maintain the model with the best quality criteria for the adjustment and did not include FEV_1_ as a possible proxy for severity of COPD in the model.

## Conclusions

This large report of ACOS in Latin America indicates that the variability in the ACOS prevalence is clearly linked with the definitions used for asthma and COPD, and the population being studied. The spirometry plus symptom-based (wheezing plus reversibility and FEV_1_/FVC <0.70) definition identifies a clinical phenotype with more frequent exacerbations, which is probably associated with a different management and treatment approach. Further evidence, including prospective longitudinal studies focusing in the validation of the diagnostic criteria with more standardised outcome measures, is clearly needed to clarify the burden of this disease.

## Additional file

Additional file 1: Table S1. Prevalence ratio and relative risk (crude and adjusted analyses for all variables in the model + FEV^1^) for exacerbations, hospitalisations due to exacerbation in the past year and mMRC scale in the different phenotypes. The regression coefficient crude and adjusted analyses for all variables in model * + FEV_1_ (absolute values, ml), for all variables in model * + FEV_1_ (absolute values, ml) + height, for all variables in model * + FEV_1_ (% predicted according to PLATINO equation) and for all variables in model * + GOLD stages in the different phenotypes. (DOCX 17 kb)

## Abbreviations

ACOS: Asthma–COPD overlap syndrome
COPD: Chronic obstructive pulmonary disease
FEV_1_: Forced expiratory volume in 1 s
FVC: Forced vital capacity
GINA: Global initiative for asthma
GOLD: Global initiative for chronic obstructive lung disease
HRQOL: Health-related quality of life
ICS: Inhaled corticosteroid
