# Supplementary material for: Asthma–COPD overlap syndrome (ACOS) in primary care of four Latin America countries: the PUMA study
Source: BMC Pulm Med. 2017 Apr 21;17:69. doi: 10.1186/s12890-017-0414-6 (PMC5401386; doi:10.1186/s12890-017-0414-6)
Supplement: Additional file 1: Table S1. — Prevalence ratio and relative risk (crude and adjusted analyses for all variables in the model + FEV1) for exacerbations, hospitalisations due to exacerbation in the past year and mMRC scale in the different phenotypes. The regression coefficient crude and adjusted analyses for all variables in model * + FEV1 (absolute values, ml), for all variables in model * + FEV1 (absolute values, ml) + height, for all variables in model * + FEV1 (% predicted according to PLATINO equation) and for all variables in model * + GOLD stages in the different phenotypes. (DOCX 17 kb) [file 12890_2017_414_MOESM1_ESM.docx]

| **Supplementary Table S1. Prevalence ratio and relative risk (crude and adjusted analyses) for exacerbations, hospitalisations due to exacerbation in the past year and mMRC scale in the different phenotypes** | | | | | | |
| --- | --- | --- | --- | --- | --- | --- |
|  | | **Asthma** | **p-value** | **COPD** | **ACOS** | **p-value** |
|  | ***Asthma defined by wheezing + reversibility*** | | | | | |
| **Exacerbations in the past year (yes/no)** | |  |  |  |  |  |
| Unadjusted – PR (95% CI) | | 1.85 (0.85; 4.06) | *0.122* | 1.00 | 2.68 (1.30; 5.52) | *0.007* |
| Adjusted*– PR (95% CI) | | 2.24 (0.92; 5.45) | *0.075* | 1.00 | 2.20 (1.10; 4.39) | *0.026* |
| Adjusted**– PR (95% CI) | | 2.87 (1.18; 7.00) | *0.020* | 1.00 | 2.37 (1.21; 4.65) | *0.012* |
| Adjusted***– PR (95% CI) | | 2.59 (1.02; 6.61) | *0.046* | 1.00 | 2.40 (1.23; 4.71) | *0.011* |
| Adjusted****– PR (95% CI) | | 3.11 (1.19; 8.11) | *0.021* | 1.00 | 2.38 (1.21; 4.65) | *0.012* |
| Adjusted*****– PR (95% CI) | | 5.63 (1.42; 22.39) | *0.014* | 1.00 | 2.54 (1.31; 4.94) | *0.006* |
| **Number of exacerbations in the past year** | |  |  |  |  |  |
| Unadjusted – RR (95% CI) | | 1.77 (0.72; 4.34) | *0.210* | 1.00 | 2.10 (0.90; 4.90) | *0.086* |
| Adjusted*– RR (95% CI) | | 2.84 (0.94; 8.61) | *0.065* | 1.00 | 1.64 (0.78; 3.44) | *0.191* |
| Adjusted**– RR (95% CI) | | 4.22 (1.55; 11.49) | *0.005* | 1.00 | 1.85 (0.92; 3.76) | *0.086* |
| Adjusted***– RR (95% CI) | | 4.13 (1.40; 12.18) | *0.010* | 1.00 | 1.86 (0.91; 3.29) | *0.087* |
| Adjusted****– PR (95% CI) | | 4.82 (1.61; 14.49) | *0.005* | 1.00 | 1.85 (0.91; 3.75) | *0.087* |
| Adjusted*****– PR (95% CI) | | 9.35 (1.93; 45.21) | *0.005* | 1.00 | 1.98 (0.97; 4.04) | *0.062* |
| **Hospitalisations in the past year** | |  |  |  |  |  |
| Unadjusted – PR (95% CI) | | 0.76 (0.10; 5.96) | *0.795* | 1.00 | 2.89 (0.80; 10.39) | *0.104* |
| Adjusted*– PR (95% CI) | | 3.57 (0.48; 26.59) | *0.214* | 1.00 | 1.65 (0.53; 5.06) | *0.385* |
| Adjusted**– PR (95% CI) | | 9.89 (1.11; 87.81) | *0.040* | 1.00 | 3.63 (1.05; 12.59) | *0.042* |
| Adjusted***– PR (95% CI) | | 12.90 (1.40; 118.60) | *0.021* | 1.00 | 3.68 (1.08; 12.51) | *0.037* |
| Adjusted****– PR (95% CI) | | **Model did not converge** | | | | |
| Adjusted*****– PR (95% CI) | | 30.89 (3.29; 289.97) | *0.003* | 1.00 | 3.15 (1.03; 9.66) | *0.045* |
| **mMRC scale** | |  |  |  |  |  |
| Unadjusted – RR (95% CI) | | 0.64 (0.41; 0.99) | *0.289* | 1.00 | 1.17 (0.88; 1.56) | *0.046* |
| Adjusted*– RR (95% CI) | | 0.73 (0.48; 1.12) | *0.149* | 1.00 | 1.22 (0.92; 1.12) | *0.176* |
| Adjusted**– RR (95% CI) | | 1.08 (0.69; 1.69) | *0.723* | 1.00 | 1.24 (0.95; 1.61) | *0.112* |
| Adjusted***– RR (95% CI) | | 1.16 (0.74; 1.80) | *0.525* | 1.00 | 1.20 (0.92; 1.56) | *0.185* |
| Adjusted****– RR (95% CI) | | 1.12 (0.72; 1.74) | *0.619* | 1.00 | 1.20 (0.92; 1.56) | *0.184* |
| Adjusted*****– RR (95% CI) | | 1.37 (0.84; 2.26) | *0.210* | 1.00 | 1.23 (0.93; 1.63) | *0.141* |
|  | ***Asthma defined as medical diagnosis*** | | | | | |
| **Exacerbations in the past year (yes/no)** | |  |  |  |  |  |
| Unadjusted – PR (95% CI) | | 1.80 (1.01; 3.20) | *0.046* | 1.00 | 1.80 (0.91; 3.53) | *0.089* |
| Adjusted*– PR (95% CI) | | 1.57 (0.75; 3.27) | *0.231* | 1.00 | 1.29 (0.64; 2.60) | *0.480* |
| Adjusted**– PR (95% CI) | | 2.16 (1.01; 4.66) | *0.047* | 1.00 | 1.22 (0.60; 2.46) | *0.582* |
| Adjusted***– PR (95% CI) | | 2.09 (0.95; 4.63) | *0.069* | 1.00 | 1.23 (0.61; 2.48) | *0.568* |
| Adjusted****– PR (95% CI) | | 2.28 (0.99; 5.25) | *0.052* | 1.00 | 1.23 (0.62; 2.47) | *0.555* |
| Adjusted*****– PR (95% CI) | | 3.13 (0.98; 10.04) | *0.055* | 1.00 | 1.24 (0.62; 2.46) | *0.541* |
| **Number of exacerbations in the past year** | |  |  |  |  |  |
| Unadjusted – RR (95% CI) | | 1.92 (0.99; 3.68) | *0.054* | 1.00 | 1.68 (0.77; 3.66) | *0.191* |
| Adjusted*– RR (95% CI) | | 2.01 (0.94; 4.30) | *0.072* | 1.00 | 1.32 (0.60; 2.88) | *0.490* |
| Adjusted**– RR (95% CI) | | 3.37 (1.54; 7.40) | *0.002* | 1.00 | 1.22 (0.57; 2.60) | *0.614* |
| Adjusted***– RR (95% CI) | | 3.33 (1.49; 7.47) | *0.003* | 1.00 | 1.22 (0.57; 2.61) | *0.605* |
| Adjusted****– PR (95% CI) | | 3.52 (1.50; 8.26) | *0.004* | 1.00 | 1.24 (0.58; 2.64) | *0.574* |
| Adjusted*****– PR (95% CI) | | 5.35 (1.58; 18.11) | *0.007* | 1.00 | 1.25 (0.60; 2.61) | *0.553* |
| **Hospitalisations in the past year** | |  |  |  |  |  |
| Unadjusted – PR (95% CI) | | 0.58 (0.16; 2.16) | *0.419* | 1.00 | 1.07 (0.29; 3.92) | *0.923* |
| Adjusted*– PR (95% CI) | | 0.68 (0.17; 2.69) | *0.581* | 1.00 | 0.72 (0.21; 2.44) | *0.596* |
| Adjusted**– PR (95% CI) | | 1.47 (0.25; 8.61) | *0.669* | 1.00 | 0.72 (0.23; 2.29) | *0.576* |
| Adjusted***– PR (95% CI) | | 1.14 (0.22; 5.79) | *0.875* | 1.00 | 0.69 (0.21; 2.23) | *0.537* |
| Adjusted****– PR (95% CI) | | 1.45 (0.25; 8.57) | *0.679* | 1.00 | 0.73 (0.23; 2.36) | *0.605* |
| Adjusted*****– PR (95% CI) | | 1.35 (0.27; 6.67) | *0.715* | 1.00 | 0.71 (0.22; 2.31) | *0.567* |
| **mMRC scale** | |  |  |  |  |  |
| Unadjusted – RR (95% CI) | | 0.90 (0.72; 1.14) | *0.311* | 1.00 | 1.24 (0.98; 1.56) | *0.052* |
| Adjusted*– RR (95% CI) | | 0.97 (0.75; 1.25) | *0.799* | 1.00 | 1.20 (0.96; 1.21) | *0.108* |
| Adjusted**– RR (95% CI) | | 1.27 (0.96; 1.67) | *0.095* | 1.00 | 1.15 (0.93; 1.42) | *0.198* |
| Adjusted***– RR (95% CI) | | 1.32 (1.00; 1.75) | *0.052* | 1.00 | 1.14 (0.93; 1.41) | *0.214* |
| Adjusted****– RR (95% CI) | | 1.38 (1.04; 1.84) | *0.028* | 1.00 | 1.17 (0.94; 1.44) | *0.152* |
| Adjusted*****– RR (95% CI) | | 1.70 (1.19; 2.42) | *0.003* | 1.00 | 1.17 (0.94; 1.46) | *0.160* |

*Normal adjustment in the paper analysis

**Adjustment for all variables in model * + FEV_1_ (absolute values, ml)

***Adjustment for all variables in model * + FEV_1_ (absolute values, ml) + height

****Adjustment for all variables in model * + FEV_1_ (% predicted according to PLATINO equation)

*****Adjustment for all variables in model * + GOLD stages
